# Targeting vascular endothelial growth receptor-2 (VEGFR-2): structural biology, functional insights, and therapeutic resistance

**DOI:** 10.1007/s12272-025-01545-1

**Published:** 2025-05-08

**Authors:** Fahad Hassan Shah, Yoon Seok Nam, Jun Young Bang, In Seo Hwang, Dae Hong Kim, Minkyoung Ki, Heon-Woo Lee

**Affiliations:** 1 College of Pharmacy, Chosun University, Gwangju, Republic of Korea; 2 Institute of Well-Aging Medicare & Chosun University G-LAMP Project Group, Chosun University, Gwangju, Republic of Korea; 3 Department of Pharmacy, College of Pharmacy, Kyung Hee University, 26 Kyungheedae-ro, Dongdaemun-gu, Seoul, Korea

**Keywords:** Angiogenesis, VEGF, VEGFR-2, Signaling, Resistance, Pathology

## Abstract

Angiogenesis, the process of new blood vessel formation, is a fundamental physiological process implicated in several pathological disorders. The vascular endothelial growth factors (VEGFs) and their receptors (VEGFRs) are crucial for angiogenesis and vasculogenesis. Among them, the tyrosine kinase receptor VEGFR-2 is primarily expressed in endothelial cells (ECs). These cells regulate various physiological responses, including differentiation, cell proliferation, migration, and survival, by binding to VEGF mitogens. Vascular Endothelial Growth Factor Receptor 2 (VEGFR-2) is a key regulator of this process, making it a prime target for therapeutic intervention. Several drugs targeting VEGFR-2 have been approved and are currently utilized to halt the pathological axis of VEGF-VEGFR. This review will focus on the recent developments in the molecular structure and function of VEGFR-2, the molecular mechanism of VEGFR-2 activation, and its downstream signaling pathway. It will also discuss therapies and experimental drugs approved to inhibit the function of VEGFR-2 and the resistance mechanism.

## Introduction

Angiogenesis, the physiological process through which new blood vessels form from pre-existing vessels, plays a crucial role in health and disease. This process is fundamental to the growth and development of organisms, as well as the healing of wounds. However, it also contributes to the progression of various pathological conditions, including cancer, where it facilitates tumor growth and metastasis by supplying nutrients and oxygen. The mechanisms underlying angiogenesis are complex and highly regulated, balancing pro- and anti-angiogenic factors. These mechanisms can be divided into several key steps: activation of endothelial cells (ECs), basement membrane degradation, EC proliferation and migration, tube formation, and, ultimately, the maturation and stabilization of newly formed blood vessels (Shah and Lee 2024).

Activation of endothelial cells is typically initiated by signaling molecules such as VEGF, which binds to receptors on the surface of ECs. This binding initiates a cascade of intracellular signaling that leads to the expression of enzymes capable of degrading the basement membrane, thereby enabling the migration of endothelial cells. The migration and proliferation of endothelial cells are directed toward the source of angiogenic signals. These cells then align to form tubular structures, a process mediated by cell–cell adhesion molecules and extracellular matrix components. Finally, the new vessels mature and stabilize through pericyte recruitment and the deposition of a new basement membrane, ensuring the structural and functional integrity of the developing vasculature. The regulation of angiogenesis is a delicate equilibrium, with an array of growth factors, inhibitors, and environmental conditions influencing the process. Disruptions in this balance can lead to either excessive or insufficient angiogenesis, contributing to the pathogenesis of diseases. Therefore, understanding the mechanisms of angiogenesis is critical for developing therapeutic strategies to modulate angiogenesis in disease treatment and tissue engineering.

VEGF receptors belong to the family of tyrosine kinases that are transmembrane receptors implicated in angiogenesis and lymphangiogenesis. The activity of these receptors is regulated by key signaling molecules known as VEGF mitogens, such as VEGF A-D, which play a crucial role in vascular maintenance, development, and various pathological conditions (Ghalehbandi et al. 2023). There are three types of VEGF receptors, which include VEGFR-1, VEGFR-2, and VEGFR-3. VEGFR-1 has an affinity for VEGF-A and is involved in monocyte migration and hematopoiesis (Kaufman et al. 2021). This receptor also acts as a decoy receptor to control the availability of VEGF ligands and is expressed on the surface of monocytes, macrophages, and endothelial cells (Weddell et al. 2018). VEGFR-2 demonstrates robust tyrosine kinase activity, serving as a critical regulator of VEGF-induced angiogenesis. It is predominantly localized on the surface of endothelial cells, where it facilitates key signaling processes essential for vascular development (Shaik et al. 2020). It has a strong affinity for VEGF-A and promotes endothelial cell migration, proliferation, differentiation, and survival. On the other hand, VEGFR-3 is mainly expressed in lymphatic endothelial cells and has a strong affinity for VEGF-C and VEGF-D. It is important for lymphangiogenesis during embryonic development and is also involved in the regulation of vascular integrity and some pathological conditions, such as tumor-associated lymphangiogenesis (Korhonen et al. 2022).

However, this review article aims to summarize the molecular structure and function of VEGFR, with a particular focus on VEGFR-2, its molecular activation, and the signaling pathways it mediates. Additionally, it discusses the role of VEGFR-2 in the pathophysiology of angiogenesis-related diseases, therapies approved for treating VEGFR-2-regulated pathological angiogenesis, and associated resistance mechanisms.

## Structure and function of VEGFR-2

VEGFR is an essential receptor tyrosine kinase implicated in regulating angiogenesis and vasculogenesis. This receptor is a central part of the VEGF-VEGFR system that is crucial to both pathological and physiological angiogenesis and has been explored in biomedical research and the development of therapeutics. The structure and function of VEGFR-2 have been highlighted below:

### Molecular characteristics of VEGFR-2

The kinase insert domain receptor (KDR) gene is located on chromosome 4q11-12. This gene encodes human VEGFR-2, a transmembrane glycoprotein composed of 1,356 amino acids (Modi and Kulkarni 2022). VEGFR-2 exists in three different forms classified by molecular weight: the glycosylated mature form (230 kDa) (Takahashi and Shibuya 1997), the non-glycosylated form (150 kDa), and an intermediate form (200 kDa) (Shen et al. 1998). However, only the mature glycosylated form is implicated in intracellular signal transduction. In contrast, other forms are either less active or in the process of becoming fully active, as N-glycosylation is necessary for the receptor’s folding, stability, and cell surface expression (Chandler et al. 2019). In mice, the structure of VEGFR-2 consists of 1,367 amino acids and exists in three structural forms with molecular weights of 180 kDa, 200 kDa, and 220 kDa. It shares 83% structural similarity with human VEGFR-2 (Wang et al. 2020). This receptor is mainly found on the surface of vascular endothelial cells, lymphatic endothelial cells, and embryonic precursor cells, including megakaryocytes, and hematopoietic stem cells (Katoh et al. 1995; Holmes et al. 2007). VEGFR-2 binds to ligands such as VEGF-A, VEGF-C, and VEGF-D, which activate the receptor to mediate the proliferation, invasion, migration, and survival of endothelial cells, while also promoting neovascularization and increasing vascular permeability (Katoh et al. 1995).

### Molecular structure of VEGFR-2

**Fig. 1:**
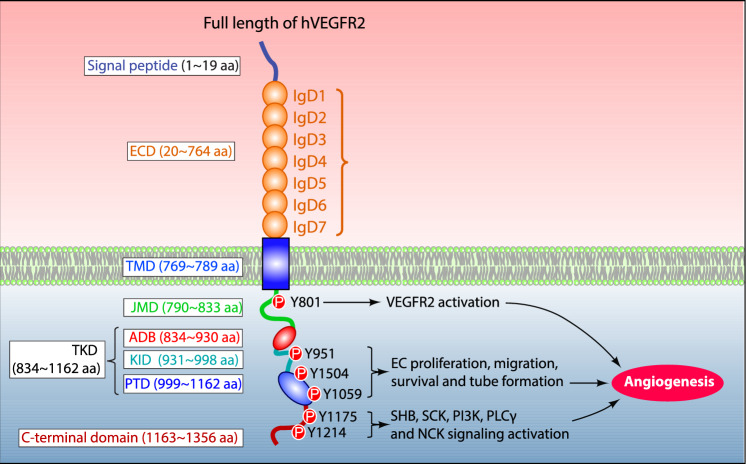
The molecular illustration of human VEGFR-2 structure. The VEGFR-2 structure is comprised of a signal peptide, an extracellular domain containing seven immunoglobulin-like subdomains (IgD1-7), transmembrane-, juxtamembrane domain (JMD), a catalytic tyrosine kinase domain composed of ATP binding (ADB)-, kinase insert domain (KID) and phosphotransferase domain (PTD) as well as C-terminal domain. Among these domains, key tyrosine residues are phosphorylated upon binding of VEGF to VEGFR2, such as Tyr801 implicated in VEGFR2 activation. Tyr951, Tyr1504, and Tyr1059 located within KID and PTD, are involved in ECs proliferation, migration, and tube formation. The residues present in the c-terminal domain (Tyr1175 and Tyr1214) activate SHB-SCK-PI3 K, PLCγ and NCK signaling pathways which are essential for angiogenesis

The human VEGFR-2 structure is comprised of 1356 amino acids that are divided into a mature protein (20–1356 aa) and a signal peptide (1–19 aa) (Park et al. 2018; Wang et al. 2020) (Fig. 1). The mature transmembrane protein is further segregated into several structural domains that facilitate its function: A Signal peptide (1–19 aa), extracellular domain (20–764 aa; ECD), transmembrane domain (765–789 aa; TMD), juxtamembrane domain (790–833 aa; JMD), ATP binding domain (834–930 aa; ADB), kinase insert domain (931–998 aa; KID), phosphotransferase domain (999–1162 aa; PTD) and flexible c-terminal domain (1163–1356 aa) (Wang et al. 2020). The mature human VEGFR-2 protein contains 15 phosphorylation sites, including several substrates and ATP binding sites and 18 N-linked glycosylation sites that are essential for VEGFR-2 post-translational modifications, cellular attachment, protein folding, and activation to modulate VEGFR-2 functions (Croci et al. 2014; Chandler et al. 2019; Chung et al. 2019). (Fig. 1).

#### Extracellular domain (ECD: 20–764 amino acids)

The ECD is further subdivided into 7 immunoglobulin-like subdomains, such as IgD1 (46∼110 aa), IgD2 (141∼207 aa), IgD3 (224∼320 aa), IgD4 (328∼414 aa), IgD5 (421∼548 aa), IgD6 (551∼660 aa), and IgD7 (667∼753 aa) (Fuh et al. 1998). All these subdomains are linked together by the linkers and possess 18 glycosylation sites (Croci et al. 2014). The function of these subdomains is to control the receptor binding to ligands primarily to VEGF-A, C, and D, unbinding from the ligands and receptor dimerization, including following autophosphorylation of the intracellular tyrosine kinase domain as reviewed extensively by Wang et al. 2020 and Park et al. 2018 (Park et al. 2018; Wang et al. 2020).

#### Tyrosine kinase domain (TKD: 834–1162 amino acids)

The tyrosine kinase domain consists of three subdomains: ATP binding domain (ADB), a phosphotransferase domain (PTD), and a kinase insert domain (KID) (Koch et al. 2011). (Fig. 1) Among VEGFRs, TKD is the conserved region that constitutes a two-lobed spatial structure that allows both lobes to form an active center between them. There is a hydrophobic pocket present at the intracellular N-terminus of the tyrosine kinase domain harboring a glycine-rich (GXGXXG, 841∼846 aa) motif and ATP-phosphate binding loop in their β-sheet structures (McTigue et al. 2012). Whereas, the c-terminus of the tyrosine kinase domain contains several α-helical structures containing an activation loop (A-loop, 1045∼1075 aa) and a catalytic loop (HRDLAARN, 1026∼1033 aa) that perform catalytic functions of VEGFR-2 (McTigue et al. 2012).

#### Transmembrane domain (TMD: 765–789 amino acids) and juxtamembrane domain (JMD: 790–833 amino acids)

The TMD regulates the kinase activity of VEGFR-2, facilitating specific orientations of intracellular domains and promoting the dimerization of receptor monomers (Koch et al. 2011; Koch and Claesson-Welsh 2012). The JMD is a single transmembrane domain that has an α-helical structure and is implicated in regulating the rate of auto-phosphorylation of VEFGR2 (Solowiej et al. 2009). (Fig. 1).

#### Carboxy terminal domain (1163-1356 amino acids)

This domain has two autophosphorylation sites that are critical for the activation and signaling of VEGFR-2 and also have a role in endothelial cell proliferation (Sase et al. 2009). It also mediates cellular signaling, cell migration, and permeability of vascular endothelial cells (Koch et al. 2011; Koch and Claesson-Welsh 2012; Manni et al. 2014).

### Molecular activation of VEGFR-2

The ligand binding/interaction of VEGF-A, -C, and -D with the VEGFR-2 receptor mediates a cellular signaling pathway that leads to homo and heterodimerization followed by phosphorylation of tyrosine residues within the receptor. VEGFR-2 dimerization attracts different signaling molecules that modulate downstream signaling pathways that affect the physiological properties of the entire vascular environment and endothelial cells (Ma et al. 2011). As previously described, the structure of VEGFR-2 contains a vital domain necessary for VEGFR-2 dimerization known as ECD. This domain is composed of various immunoglobulin-like subdomains connected via linkers and contains multiple glycosylation sites. Each monomer of VEGF is made up of five β-sheets and two α-helix that construct an antiparallel β-sheet. These VEGF monomers contain a specialized structural motif called a cysteine knot. These knots signify intramolecular disulfide bridges formed between specific pairs of cysteine amino acids within the VEGF-A protein: Cys263-Cys308, Cys267-Cys310, and Cys232-Cys274 that aid in stabilizing their three-dimensional structure necessary for its biological activity (Wang et al. 2020). VEGF-A has two receptor sites that bind to the VEGFR-2 receptor. Moreover, it also has dimerization interface sites that facilitate the dimerization of VEGFR-2 receptors. These ligands bind to the IgD2 and IgD3 subdomains of ECD of VEGFR-2, whereas Ig-like subdomains 4–7 (IgD4-7) are necessary for the VEGF-mediated VEGFR activity and stabilization of VEGF-bound VEGFR-2 dimers (Yang et al. 2010). The glycosylation sites present within the ECD of VEFGR-2 play a vital role in ligand binding, stabilizing, trafficking, and pro-angiogenic signaling in pathological and physiological processes (Croci et al. 2014; Chandler et al. 2019). The kinase activity of VEGFR-2 is regulated by the juxtamembrane domain and transmembrane domain that activate VEGFR-2 function (Holmes et al. 2007; Solowiej et al. 2009). The transmembrane domain is responsible for the correct and specific orientation of receptor monomers upon VEGF-induced ECD Rearrangement to ensure proper cellular signaling (Holmes et al. 2007). Whereas the juxtamembrane domain has a crucial phosphorylation site at tyrosine residue Tyr801 (Y801) (Solowiej et al. 2009). When this residue is unphosphorylated, the kinase activity of VEFGR-2 is autoinhibited due to the interaction of the activation loop (A-loop) in the kinase domain 2 (TKD2) (Walter et al. 2007; Solowiej et al. 2009). Upon phosphorylation, the inhibitory interaction between the A-loop and the JMD is disrupted, which allows re-orientation of the A-loop to favor an enzymatically active conformation, leading to the activation of VEGFR-2 function. The subdomains of the TKD, such as the TKD1, TKD2, and the KID, are important in regulating the cellular signaling and activation of VEGFR-2. Upon VEGFs-mediated dimerization of VEGFR-2 causes the TKD transphosphorylation of several residues, including Tyr951 (Y951) in the KID domain, Tyr1054 (Y1054) and Tyr1059 (Y1059) located in the A-loop of the TKD2 to activate the kinase activity (Takahashi et al. 2001; Matsumoto et al. 2005; Sase et al. 2009). This event then regulates endothelial cell proliferation, migration, survival, and vascular tube formation in addition to cellular signaling (Holmes et al. 2007; Koch et al. 2011). Another important domain in the VEGFR-2 activation and signaling is the carboxyl terminus domain. This domain contains two vital autophosphorylation sites, Tyr1175 (Y1175) and Tyr1214 (Y1214), that are phosphorylated upon VEGF-A binding to VEGFR-2 (Sase et al. 2009). Then, several signaling molecules, including adapter proteins SHB and SCK, PI3 K, PLCγ, and NCK, bind to VEGF-activated VEGFR-2 that mediates VEGFR-2 activation and increases the migration, permeability, and proliferation of vascular endothelial cells (Warner et al. 2000; Takahashi et al. 2001; Holmqvist et al. 2004; Lamalice et al. 2006; Koch and Claesson-Welsh 2012; Manni et al. 2014).

### VEGFR-2 signaling pathways

The maintenance and development of the physiological function of different tissues and organ-specific vascular systems are essential for overall health. These functions are essentially regulated by the VEGF/VEGFR-2 signaling. This signaling is also reported in the pathogenesis of different diseases, i.e., cancer and cardiovascular diseases. It has been proven to control important cellular signal transduction, such as endothelial cell proliferation, survival, migration, and increased vascular permeability. The signaling pathways modulated by the activation of the VEGFR-2 include the PLCγ-PKC-Raf-MEK-MAPK signaling pathway (endothelial cell proliferation). PLCγ exists in two main isoforms: PLCγ1 and PLCγ2. PLCγ1 is associated with promoting endothelial cell proliferation (Sjoberg et al. 2023), while PLCγ2 appears to play a role in inhibiting this process. (Zhao et al. 2024). The TSAd-SRC-PI3 K-PKB/AKT signaling pathway (endothelial cell survival), and the NCK/SRC-p21/Cdc42-SAPK2/p38-MAPK pathway (endothelial cell migration) and vascular permeability.

#### Endothelial cell proliferation and differentiation pathway

**Fig. 2:**
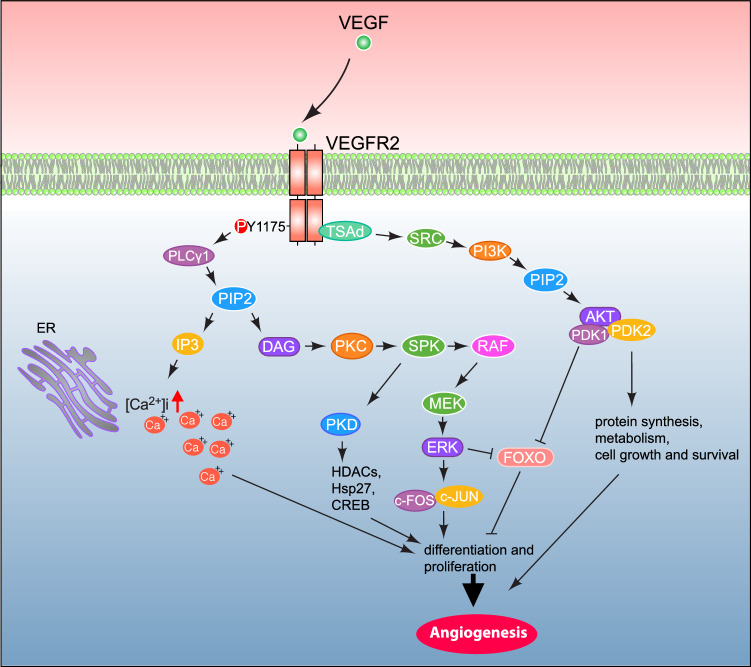
The molecular mechanism regulated by VEGFR-2 mediated angiogenesis. Upon binding of VEGF mitogens to the **VEGFR-2** receptor, it orchestrates dimerization and phosphorylation of (tyrosine Y1175) residue of the c-terminal domain that activates phospholipase C gamma (PLCγ1). PLCγ1 mediates the activation of phosphatidylinositol 4,5-bisphosphate (PIP2) that stimulates inositol trisphosphate (IP3) and diacylglycerol (DAG). Inositol trisphosphate (IP3) fosters calcium ions production whereas diacylglycerol (DAG) activates protein kinase C which in turn stimulates sphingosine kinase (SPK). SPK activates protein kinase D (PKD) and Raf protein. PKD interacts with histone deacetylases, heat shock protein 27 (HSP27), and cAMP-response-element binding protein (CREB) that aids in the process of angiogenesis whereas RAF modulates MEK and ERK to direct the endothelial cells'differentiation, and proliferation, and survival. The dimerization and phosphorylation event attracts the binding of TSAd that modulates a cascade of activation of different molecular factors such as SRC, PI3 K, PIP2, AKT, PKD1, and PKD2 that directs protein synthesis, metabolism, and cell growth and survival

When VEGF binds to the VEGFR-2 receptor, it activates the receptor, leading to dimerization and autophosphorylation of specific tyrosine subunits within the VEGFR-2 intracellular domain. This process regulates the cell proliferation and differentiation signaling pathway, which is crucial for angiogenesis, directing the signal to the nucleus to initiate DNA synthesis (Takahashi et al. 1999). Phospholipase C gamma (PLCγ) is activated upon phosphorylation of the C-terminal Y1175 residue of VEGFR-2 (Takahashi et al. 2001; Sase et al. 2009) that catalyzes phosphatidylinositol 4,5-bisphosphate (PIP2), resulting in the formation of two secondary messengers, inositol trisphosphate (IP3) and diacylglycerol (DAG), and produces a binding site to attract different signaling mediators, such as the adapter proteins SHB and SCK and SHCA and GRB2 (Warner et al. 2000; Holmqvist et al. 2004). (Fig. 2) The function of IP3 is to boost the intracellular concentration of calcium ions. At the same time, DAG’s role lies in activating PKC. DAG-activated PKC (Takahashi et al. 1999) stimulates the activation of sphingosine kinase (SPK) (Shu et al. 2002), which in turn leads to Raf kinase activation. It also involves the stimulation of other PKC family members implicated in activating protein kinase D (PKD) (Shu et al. 2002; Wong and Jin 2005). The suppression of MAPK/ERK1/2 activation upon PKD inhibition suggests that PKD contributes to the activation of this signaling pathway (Tyagi et al. 2023). The function of PKD is to assist histone deacetylases (HDACs) 5 and 7 into the nucleus. PKD also promotes the phosphorylation of heat shock protein 27 (Hsp27) and cAMP-response element-binding protein (CREB) (Wong and Jin 2005; Wang et al. 2008). Raf is a serine/threonine-specific protein kinase that activates MEK (MAPK/ERK kinase), which in turn activates extracellular signal-regulated kinase (ERK) (Shu et al. 2002). Activated ERK is then translocated to the endothelial cell nucleus to phosphorylate various transcription factors. That stimulates the gene expression implicated in cell differentiation, proliferation, and survival. (Fig. 2) Phosphorylated ERKs also activate c-FOS and c-Jun, forming the AP-1 complex, which regulates cell cycle progression genes (Bose et al. 2024). (Fig. 2) ERK-mediated c-FOS/c-Jun activation promotes transcription of angiogenesis-related genes such as TIMP1(Tissue Inhibitor Matrix Metalloproteinase-1) (Wang et al. 2024a) and MMP9 (Cheng et al. 2024), thereby enhancing tip cell behavior and neovascularization.

VEGFR-2 drives endothelial cell differentiation by triggering multiple intracellular signaling pathways indispensable for endothelial cell maturation and functionality. These pathways include MAPK/ERK and PI3 K/Akt pathways responsible for endothelial cell lineage commitment and differentiation (Wong and Jin 2005; Sase et al. 2009; Zhang et al. 2015; Tsuji-Tamura and Ogawa 2016). Collaboration between these pathways promotes cell survival, contributing to cell differentiation by regulating essential downstream gene targets, such as inactivation of FOXO factors (Zhang et al. 2015; Tsuji-Tamura and Ogawa 2016), to regulate endothelial-specific genes such as VE-cadherin, PECAM-1, vWF, eNOS, and Tie receptors. (Fig. 2) The Raf-MEK-ERK cascade, along with PLCγ/PKC, activates the activity of different transcription factors (ETS (Wei et al. 2009), Elf-1, and NFAT), aiding in differentiation (Holmes et al. 2007; Koch et al. 2011; Koch and Claesson-Welsh 2012). The expression of several ETS family transcription factors—namely ETS1, ETS2, ERG, ELK3, FLI1, ETV1, ETV2, ETV5, and ETV6—was significantly upregulated during the process of endothelial differentiation which is critical for the proper formation of vascular network with arterial, venous and capillary ECs (McCracken et al. 2023; Li et al. 2024b; Nornes et al. 2025).

#### Endothelial cell survival pathway

Angiogenesis and vascular endothelial cell survival are regulated by VEGFR-2 via the activation of the TSAd-SRC-PI3 K-PKB/AKT pathway. (Fig. 2) Dimerization and phosphorylation of VEGFR-2 attract the adaptor protein TSAd, which in turn activates Src family kinases (SFKs) (Sun et al. 2012). PI3 K is activated either through SRC or directly by TSAd. PI3 K catalyzes the phosphorylation of membrane-bound PIP2 to PIP3, which in turn activates AKT by facilitating the binding of PIP3 to the pleckstrin homology domain of AKT, along with PDK1 and PDK2 (Cantley 2002; Downward 2004). AKT then phosphorylates various cellular molecules that regulate processes such as protein synthesis, apoptosis pathways (including Rab14, ADAM10, R-Ras, and caspases 3, 7, and 9) (Herrera and Komatsu 2024; Baek et al. 2025), transcription factors, as well as cell growth and survival regulation and metabolism (Giri et al. 2024; Wang et al. 2024b). (Fig. 2)

#### Vascular permeability pathway

**Fig. 3:**
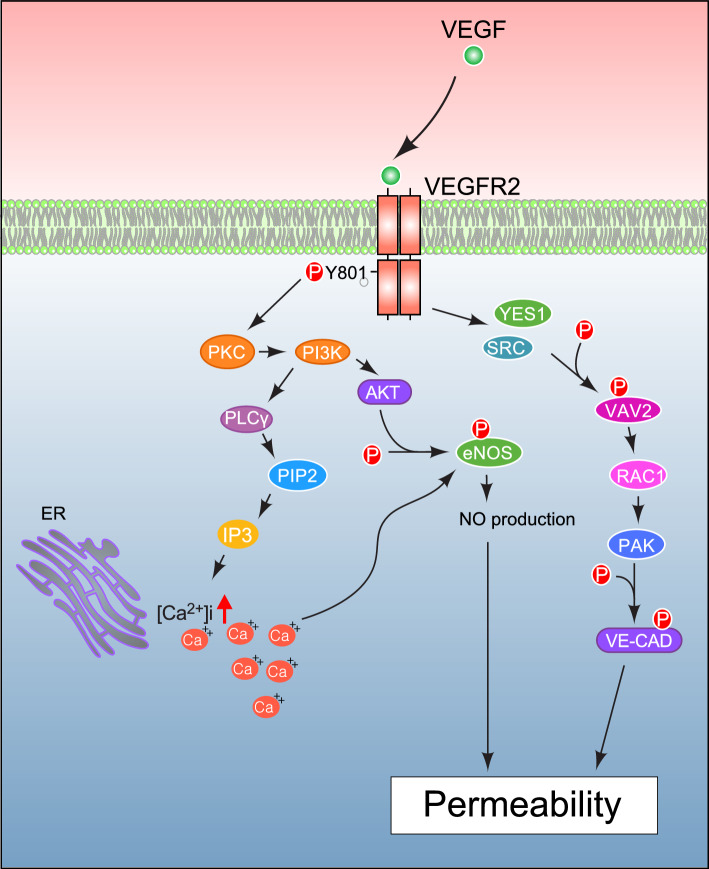
Molecular Depiction of VEGFR-2 role in Vascular Permeability of Endothelial cells. The binding of VEGF mitogen on VEGFR-2 receptor facilitates dimerization and phosphorylation of Tyrosine 801 (Y801) activates protein kinase C (PKC) that stimulates PI3 K and AKT pathway that results in the production of nitric oxide species (NO species) which in turn activate several other molecular factors such as PLCγ, PIP2 and IP3 and Ca^2+^ ions. Activated VEGFR-2 stimulates the activity of SRC and tyrosine-protein kinase (YES) that phosphorylates guanine nucleotide exchange factor (GEF) (VAV2) that activates RAC1 which then turns on p21 activated kinase (PAK) activity. PAK protein modulates VE-cadherin phosphorylation, increasing the vascular permeability of endothelial cells in VEGFR-2-mediated angiogenesis

Vascular permeability permits the blood vessels to exchange and pass molecules and cells between the bloodstream and surrounding tissues, which is achieved by two major mechanisms: the transient opening of paracellular endothelial junctions and the creation of transcellular endothelial pores (Garrido-Urbani et al. 2008). However, the signal transduction clarifying these mechanisms has not been reported yet and requires further investigation. Blanes et al. (2007) described the PKC-PI3 K/Akt pathway as one of the vital pathways that enhance vascular permeability. The phosphorylation of Tyr801 of VEGFR-2 activates endothelial nitric oxide synthase (eNOS) mainly by the PKC-PI3 K/AKT pathway. This pathway promotes and expedites NO release by endothelial cells by then binding to its molecular chaperone heat shock protein (Hsp90) (Duval et al. 2007). Whereas another mechanism of eNOS activation is AKT-mediated phosphorylation at S1179 of eNOS or PLCγ-dependent Ca^2+^ influx (Dimmeler et al. 1999; Fulton et al. 1999). Certain kinases, such as SRC and tyrosine-protein kinase YES1 (YES Proto-Oncogene 1), are also involved in the direct or indirect phosphorylation of adherent junction protein (VE-cadherin) mediated by VEGFA to regulate endothelial junctional plasticity and barrier integrity (Jin et al. 2022; Sjoberg et al. 2023; Pal et al. 2025). Dimerized and activated VEGFR-2 stimulates SRC family kinases that phosphorylate a guanine nucleotide exchange factor (GEF) VAV2 (Gavard and Gutkind 2006). VAV2 activates RAC1, which then activates p21-activated kinase (PAK) that mediates VE-cadherin phosphorylation, leading to disassembly of VE-cadherin junctions to increase vascular permeability (Fig. 3) and also reorganization of the actin cytoskeleton and cell motility.

#### Endothelial cell migration pathway

**Fig. 4:**
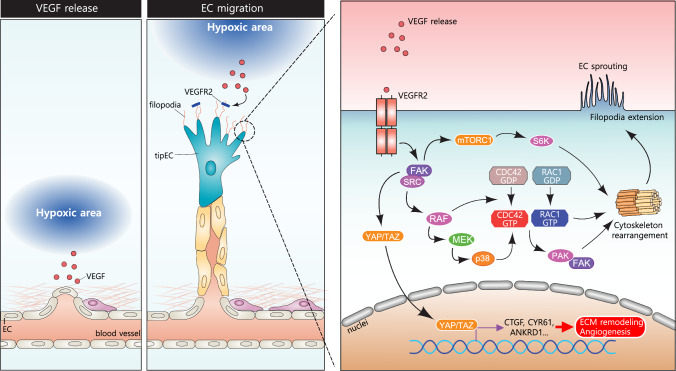
Molecular pathway controlling endothelial migration through VEGF signaling. As described in the first illustration, a hypoxic area releases VEGF mitogens that prepare the EC cells for migration. In the second illustration, EC cells proliferation and sprouting begin, which follows toward the hypoxic region where tip endothelial cells (tipECs) allow the binding of VEGF to their VEGFR2 receptor through filopodial protrusions. Within these protrusions, VEGF released from the hypoxic region binds to the VEGFR2 causing the stimulation of SRC and FAK that leads to the activation of different molecular factors such as (RAF, MEK, p38). These factors influence the activity of CDC42, RAC1, and FAK leading to cytoskeletal rearrangement resulting in EC sprouting and filopodial extension, and ultimately EC migration

Hypoxia serves as a potent stimulus for angiogenesis by inducing the expression of VEGF. Under hypoxic conditions, VEGF is secreted by various cell types, creating a chemotactic gradient that directs endothelial migration toward areas of hypoxia. This process is initiated when VEGF binds to its primary receptor, VEGFR-2, triggering receptor dimerization and phosphorylation. These events activate downstream signaling cascades essential for cytoskeletal remodeling and cell motility. (Fig. 4).

Focal adhesion kinase (FAK), a central mediator of cell adhesion and migration, is rapidly activated following VEGFR-2 stimulation (Lee et al. 2017). This activation facilitates the recruitment of SRC family kinases promoting the assembly of focal adhesion complexes (Chen et al. 2024a). These complexes serve as signaling hubs, linking the extracellular matrix (ECM) to the cytoskeleton and enabling dynamic interactions required for efficient EC migration. FAK signaling is critical for cytoskeletal rearrangement, particularly through the activation of Rho family small GTPases, including CDC42 and RAC1, and their downstream effectors, which are essential for actin cytoskeletal dynamics and cell migration. (Fig. 4) FAK influences RAC1 activation by promoting the exchange of GDP for GTP, leading to the formation of lamellipodia, which are essential for cell movement (Chen et al. 2024a). Similarly, FAK activates CDC42, which directs filopodia formation at the leading edge of migrating cells. Both RAC1 and CDC42 regulate actin polymerization and cell polarity, but RAC1 primarily facilitates broad lamellipodial protrusions, while CDC42 generates narrower, finger-like projections (Knezevic et al. 2009; Mehidi et al. 2019). (Fig. 4) Additionally, FAK can activate the SRC-RAF-p38 MAPK pathway, which further regulates CDC42 and contributes to actin filament rearrangement, focal adhesion turnover, and stress fiber formation (Zebda et al. 2012). Moreover, FAK’s interaction with p21-activated kinase (PAK), a downstream effector of RAC1 and CDC42, amplifies their effects on the tip cell positioning, promoting filopodia formation and directional migration (Nan et al. 2023; Hiepen et al. 2025). FAK is also implicated in regulating transcriptional responses vital for EC migration and angiogenesis, in addition to its role in cytoskeletal remodeling.FAK stimulates the activity of transcriptional co-activators YAP and TAZ (Hooglugt et al. 2021), promoting their nuclear translocation and subsequent regulation of target genes such as CTGF, CYR61, and ANKRD1. These target genes are crucial for ECM remodeling and angiogenesis (Li et al. 2023). Additionally, the mTORC1-S6 K pathway is involved in FAK-mediated cytoskeleton rearrangement (Dodd et al. 2015). mTORC1-mediated S6 K activation facilitates cytoskeletal reorganization in ECs by influencing the dynamics and integrity of actomyosin and focal adhesions (Arora et al. 2022). Through these coordinated actions, FAK ensures the proper orchestration of cell adhesion, protrusion formation, and migration, which are vital for processes in endothelial migration. (Fig. 4).

## Role of VEGFR-2 in angiogenesis

The role of VEGFR-2 has been greatly explored in both physiological and pathological contexts that are discussed in the following headings:

1. **Physiological functions:** VEGFR-2 plays a vital role in mediating different physiological processes rendered by the activity of VEGFs. VEGFR-2 is involved in the regulation of angiogenesis. VEGFR-2 is activated upon binding of VEGF mitogens that trigger downstream signaling pathways, stimulating endothelial cell survival, migration, and proliferation. This pathway is essential for forming new blood vessels. VEGFR-2 activation facilitates embryonic development by expanding the vascular network that supplies nutrients and oxygen to developing tissues mediated by VEGFA, promoting endothelial cell differentiation into mature blood vessels, acting as chaperons of vascular structure organization throughout the embryo, ensuring adequate vascularization, and regulating morphogenetic processes contributing to organogenesis and tissue patterning (Wang et al. 2020). Angiogenesis mediated by VEGFR-2 is necessary for wound healing by directing the new blood vessel formation at the site of injury and facilitating an adequate supply of oxygen and nutrients imperative for tissue regeneration and repair (Zhang et al. 2017). In the female reproductive cycle, VEGFR-2 plays a significant role in the development of the ovarian follicle, corpus luteum, and placenta to support reproductive efficiency and ensure adequate blood supply to the fetus (Douglas et al. 2009). Hematopoiesis is the process by which new blood cells are formed in the bone marrow. VEGFR-2 plays a role in hematopoiesis by regulating the survival and proliferation of various blood cell lineages by activating anti-apoptotic pathways and influencing their differentiation (Wang et al. 2020). During stress or injury, VEFGR-2 causes the mobilization of hematopoietic stem cells from the bone marrow into circulation (O'Donnell et al. 2016).
2. **Angiogenic switch and pathological functions:** Angiogenic switch refers to the transition of the tissue or organ towards a state characterized by increased angiogenesis involving complex molecular pathways influenced by different factors that include hypoxia, inflammation, and tissue injury (Eelen et al. 2020). There is a homeostatic balance between angiogenic and anti-angiogenic signals under normal conditions to regulate various physiological functions. However, in a pathological state, an aberration in the VEGFR-2 functioning leads to the dysregulation of the angiogenic switch, causing the onset of different pathological conditions such as cancer (Eelen et al. 2020), inflammation (Szade et al. 2015), blood vessel diseases (Salven et al. 2002), and neurological disorders (Shim and Madsen 2018). Tumor cells often overexpress VEGF mitogens that bind to VEGFR-2 on the endothelial cells, causing VEGFR-2-mediated angiogenesis. This aids metastasis and tumor growth by supplying cancerous tumors with an uninterrupted supply of oxygen and stimulates the formation of new blood vessels that infiltrate the tumor mass. VEGFR-2 activation promotes inflammatory processes by enhancing vascular permeability and inflammatory cell recruitment, contributing to different joint and vision-related impairments as seen in rheumatoid arthritis and ophthalmic diseases such as macular degeneration and diabetic retinopathy (Le and Kwon 2021). Pathological angiogenesis is caused by the overactivity of VEGFR-2 that affects tissue perfusion and blood flow regulation (Mariotti et al. 2021). In different neurological disorders, dysregulated VEGFR-2 signaling disrupts neurovascular integrity and cerebral blood flow, resulting in the neurodegeneration of vital neural tissues (Sharma et al. 2022).

## The role and mechanisms of VEGFR-2 in diverse diseases

VEGFR-2 is intimately associated with numerous diseases, including rheumatoid arthritis, diabetic retinopathy, malignant tumors, and age-related macular degeneration (AMD). Its abnormal expression, particularly in various cancers such as breast, gastric, ovarian cancer, and adult neuroblastoma, underscores the significance of VEGF/VEGFR and notch signaling pathways in both physiological and pathological angiogenesis.

### Cancer

**Fig. 5:**
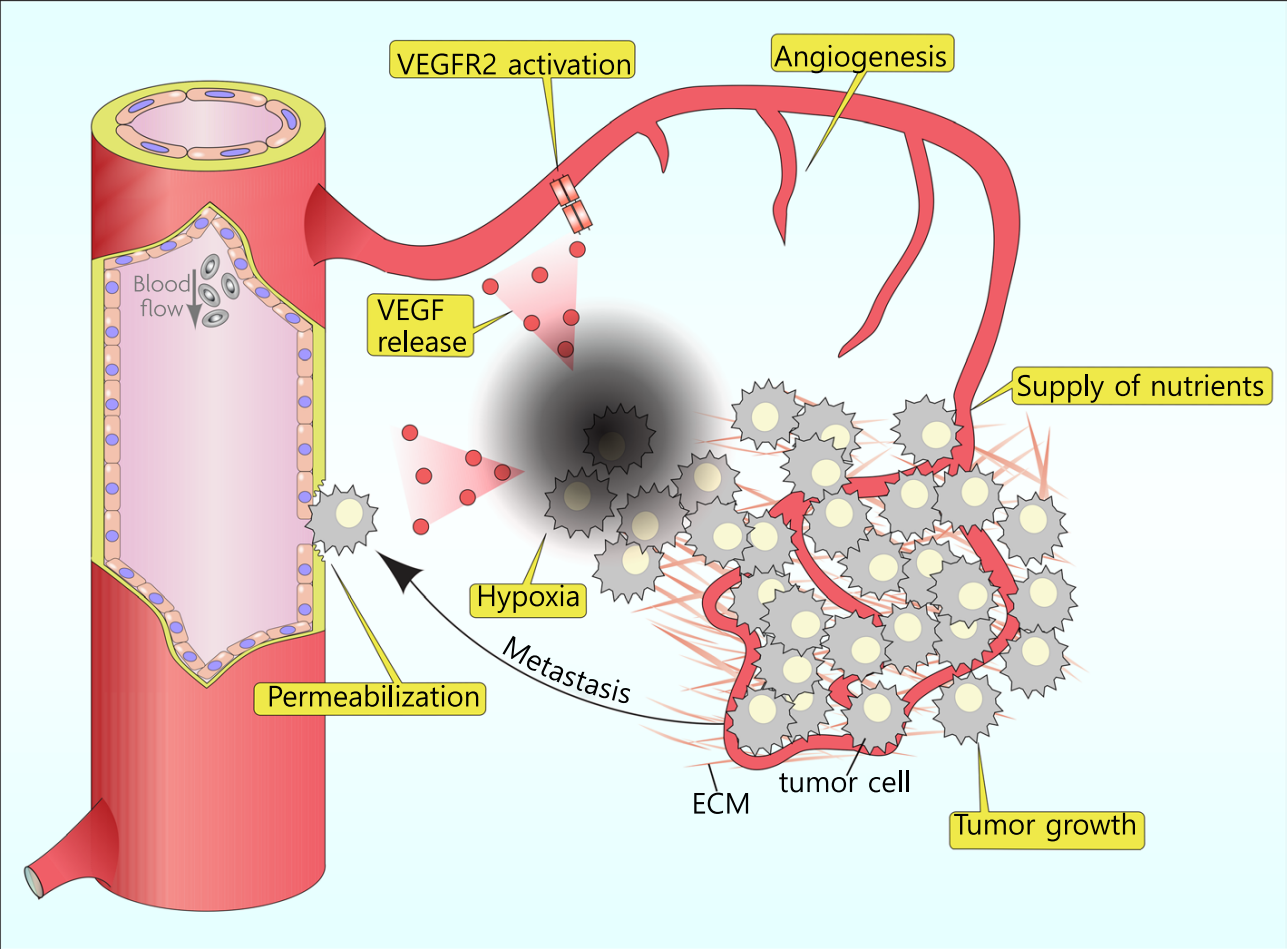
Tumor growth and VEGF-mediated angiogenesis. In response to hypoxia, tumor cells release VEGF, which activate VEGFR2. This activation initiates angiogenesis, providing essential nutrients and oxygen to support tumor growth. In addition to promoting angiogenesis, VEGFR2 activation increases blood vessel permeability, facilitating tumor metastasis

Under hypoxic conditions in the tumor microenvironment, stabilized HIF-1α upregulates VEGF expression, driving angiogenesis and enabling further tumor growth and progression. VEGF-mediated VEGFR-2 signaling plays a pivotal role in cancer progression primarily through its involvement in angiogenesis, tumor growth, and metastasis, as it supplies the tumor with oxygen and nutrients while removing waste products. (Fig. 5) Upon binding to VEGF-A, VEGFR-2 undergoes dimerization and autophosphorylation, activating several downstream signaling pathways that promote endothelial cell proliferation, migration, and survival (Sarabipour et al. 2016). These processes are essential for the formation of new blood vessels within the tumor microenvironment, facilitating tumor growth beyond a size that can be supported by diffusion alone. (Fig. 5) VEGFR-2 signaling also increases vascular permeability, allowing cancer cells to access the bloodstream more easily (Tomita et al. 2021). This effect not only contributes to the supply of nutrients to the tumor but also facilitates the escape of cancer cells from the primary tumor site, a crucial step in the metastatic process. Enhanced drug delivery upon inhibition of excessive vascular permeability indicates that increased permeability may disturb effective cancer treatment (Hoffmann et al. 2024). The activation of VEGFR-2 influences the tumor microenvironment in ways that favor cancer cell survival and spread (Zhao et al. 2022). For example, the formation of abnormal, leaky vessels due to excessive VEGFR-2 signaling can lead to hypoxia and acidosis within the tumor (Majidpoor and Mortezaee 2021). These conditions can select more aggressive cancer cells that are adept at surviving in adverse environments. Furthermore, the new vessels provide routes for cancer cells to metastasize to distant organs (Pereira et al. 2018). VEGFR-2 signaling affects not only endothelial cells but also interacts with other cell types within the tumor microenvironment, such as immune cells (Zhao et al. 2022). It can modulate the immune response to the tumor, often creating an immunosuppressive environment that allows the tumor to evade immune detection and destruction. Therefore, VEGFR-2 is a key mediator of angiogenesis in cancers, promoting tumor growth, vascular permeability, and metastasis through its interaction with VEGF-A and other components of the tumor microenvironment. Targeting VEGFR-2 has become a fundamental strategy in the fight against cancer, offering a pathway to inhibit tumor progression and improve patient outcomes.

### Diabetic retinopathy (DR)

**Fig. 6:**
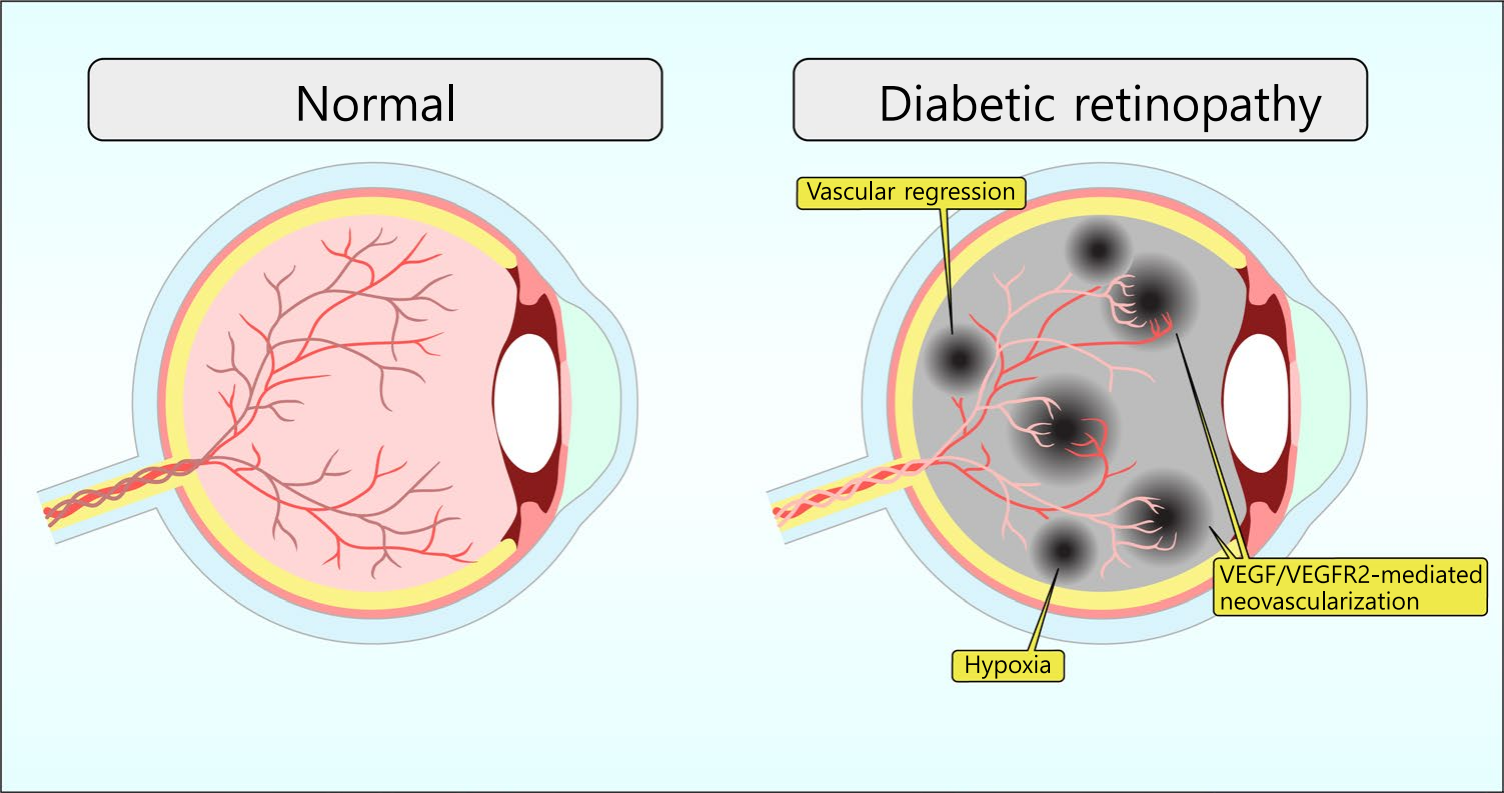
Diabetic Retinopathy (DR) and VEGF-Mediated Angiogenesis: In the healthy state (left), the retinal blood vessels are structurally stable, ensuring adequate perfusion and minimal disruption. By contrast, diabetic retinopathy (right) is marked by the regression of preexisting vessels, resulting in diminished blood flow and subsequent hypoxia. This hypoxic environment upregulates the expression of vascular endothelial growth factor (VEGF), which binds to VEGF receptor 2 (VEGFR2), promoting aberrant neovascularization

In diabetic retinopathy, chronic hyperglycemia injures the retinal microvasculature, resulting in vascular regression, microaneurysms, increased vascular permeability (leading to edema), and capillary occlusions. As these pathological changes accumulate, certain regions of the retina become hypoxic, which stimulates the expression of vascular endothelial growth factor (VEGF). The ensuing abnormal neovascularization is a critical driver of progressive vision impairment (Forrester et al. 2020). (Fig. 6) Among the various molecular pathways implicated in its pathogenesis, VEGF and its receptors (VEGFRs) play pivotal roles. VEGF has emerged as a central player in the pathogenesis of diabetic retinopathy, primarily through its interaction with VEGF receptors (Ahmad and Nawaz 2022). In particular, VEGFR-2 plays a crucial role in the angiogenesis and vascular permeability associated with DR. VEGFR-2-mediated signaling contribute to excessive neovascularization and increased vascular permeability, which are key characteristics of advanced DR. (Fig. 6) Additionally, the interaction between VEGFR-2 and ARNO activates ARF6, which stimulates VEGFR-2 internalization, while VEGFR-2 interaction with GEP100 activates ARF6 to promote VEGFR-2 recycling through coreceptor binding (Hu et al. 2020). Targeting these pathways to inhibit VEGFR-2 signaling output is effective. Research has demonstrated that ARF6 plays a vital role in VEGFR-2 trafficking. Thus, targeting ARF6-mediated VEGFR-2 trafficking offers potential therapeutic benefits for treating vascular diseases in diabetic retinopathy. Furthermore, the VEGFR-2 signaling pathway intersects with other critical pathways involved in DR, such as insulin receptor pathways (Senthil et al. 2002), inflammatory responses (Rezzola et al. 2021), and oxidative stress pathways (Caldwell et al. 2005), exacerbating retinal vascular dysfunction (Caldwell et al. 2003). Recently, Yang and Li (2023) reported that tricin has been shown to inhibit VEGFR-2 signaling by reducing ROS production in endothelial cells and decreasing VEGF expression by suppressing hypoxia-inducible factor-1α (HIF-1α) accumulation in tumor cells and damage.. This highlights the importance of discovering new therapeutic targets and treatments for DR.

### Age-related macular degeneration (AMD)

**Fig. 7:**
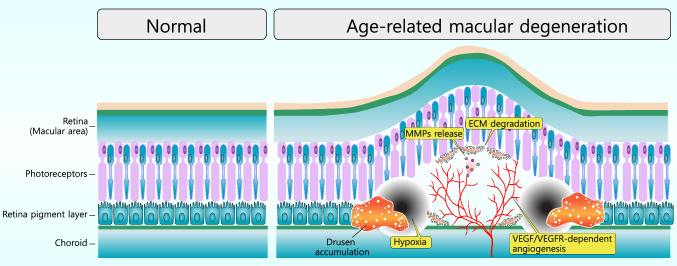
Age-related macular degeneration (AMD) and VEGF-mediated angiogenesis. In the normal macula (left), photoreceptors, the retinal pigment epithelium (RPE), and the choroid are well-organized, supporting stable visual function. By contrast, AMD is characterized by the accumulation of drusen beneath the RPE (right), which disrupts the extracellular matrix (ECM) and elevates matrix metalloproteinase (MMP) activity. The ensuing hypoxia enhances the production of vascular endothelial growth factor (VEGF), thereby activating VEGF receptor-dependent angiogenesis. These newly formed vessels are structurally fragile, leading to leakage and hemorrhage that ultimately compromise macular integrity and central vision

VEGFR-2-mediated angiogenesis plays a pivotal role in the formation of choroidal neovascularization (CNV) by driving key processes in vascular endothelial cells, particularly in the context of drusen accumulation. Drusen, yellowish deposits of lipids and proteins beneath the retinal pigment epithelium (RPE), contribute to a pro-inflammatory and hypoxic environment that stimulates the overexpression of VEGF. (Fig. 7) VEGF binds to VEGFR-2 on endothelial cells, activating downstream signaling pathways such as PI3 K/AKT and MAPK, which promote endothelial cell proliferation, migration, and survival. This signaling also increases vascular permeability via nitric oxide production. Additionally, VEGFR-2 activation induces the production of matrix metalloproteinases (MMPs), degrading extracellular matrix components and facilitating endothelial cell invasion into surrounding tissues. (Fig. 7) These processes, exacerbated by the inflammatory milieu associated with drusen, result in aberrant blood vessel growth beneath the retina, a hallmark feature of neovascular AMD (Chaudhuri et al. 2023).

Anti-VEGF therapies, such as ranibizumab (Chong 2016) and aflibercept (Sarwar et al. 2016), have revolutionized the management of neovascular AMD by inhibiting VEGF-mediated angiogenesis and reducing disease activity. However, challenges remain, including the need for sustained treatment and the emergence of resistance. Among these, research is ongoing on treatments using BetP-based hydrogel (BetP-Gel)to reduce side effects from repeated injections by allowing long-term drug release in the body, thus reducing the frequency and dosage of administration (Gao et al. 2023). VEGFR signaling pathways play a vital role in the pathogenesis of age-related macular degeneration, particularly in the development of choroidal neovascularization. Targeting VEGFR signaling represents a promising therapeutic approach for AMD, although further research is needed to optimize treatment strategies and address emerging challenges.

### Other diseases

Rheumatoid arthritis (RA) is a chronic autoimmune disorder marked by persistent inflammation and joint damage, profoundly affecting patient quality of life (Jahid et al. 2023). Among the various factors implicated in RA pathogenesis, VEGFR–2–mediated abnormal angiogenesis in the synovium is increasingly seen as pivotal (Paradowska-Gorycka et al. 2019). This process supplies essential oxygen and nutrients to inflamed areas and facilitates immune cell infiltration, perpetuating chronic inflammation. VEGF acts as a chemoattractant for inflammatory cells such as monocytes and macrophages, and signal transduction through VEGFR-2 enhances their recruitment, exacerbating synovitis and joint destruction. Moreover, VEGFR-2 interacts with inflammatory cytokines and growth factors (e.g., TNF-α and IL-1) (Paleolog et al. 1998; Chen et al. 2023), potentially producing synergistic effects that drive both angiogenesis and inflammation. VEGF binding to VEGFR-2 leads to autophosphorylation at tyrosine residues, including Tyr951, activating key intracellular pathways such as PI3 K/AKT, which is crucial for vascular endothelial cell function. Targeting this VEGFR-2/PI3 K/AKT axis is thus regarded as a strategic approach to inhibiting synovial angiogenesis in RA, and CPD-002 is currently being studied as a potential inhibitor of this pathway (Jiang et al. 2024). However, the direct role of VEGF and VEGFR in regulating RA remains incompletely understood.

In ischemic stroke, which arises from disruption or occlusion of cerebral microcirculation due to factors such as thrombosis or insufficient blood supply, VEGFR-2 promotes endothelial cell growth and migration to reconstruct damaged vessels, thereby restoring oxygen and nutrient delivery to brain tissue (Maida et al. 2020). Reduced levels of VEGFR-2 observed in PTX3 knockout mice after stroke suggest that decreased VEGF may impair this reparative process, while VEGFR-2 activation helps preserve neuronal viability and spur regeneration (Rodriguez-Grande et al. 2015). Additionally, VEGFR-2 fosters collateral vessel development, alleviating tissue ischemia, and directly supports neuron and Schwann cell function (Lange et al. 2016). However, its regulation can vary at different stages of stroke: in early ischemia, elevated vascular permeability may exacerbate edema, whereas in later phases, enhanced vessel and neuronal repair dominate (Li et al. 2011). Due to the association between VEGFR-2 and ischemic stroke, research is being conducted on the impact of BMSC-EVs and BEC-EVs on the integrity of the blood–brain barrier after acute ischemic stroke (Li et al. 2024a).

Pulmonary hypertension (PH), another serious condition, is characterized by pulmonary vascular dysfunction and right heart failure, frequently associated with high VEGF-A/VEGFR-2 expression (Zhou et al. 2022). Excessive vascular cell proliferation, vessel wall thickening, and elevated pulmonary artery pressure lead to a poor prognosis if untreated. Although VEGF-A binding to VEGFR-2 typically promotes endothelial survival and function, inhibiting this pathway in severe PH models, such as via SU5416, can paradoxically worsen outcomes by inducing endothelial cell death and allowing apoptosis-resistant cells to proliferate (Abhinand et al. 2016). Notably, selectively blocking the Y949 signaling axis of VEGFR-2 in a mouse model reduces vascular leakage, macrophage infiltration, and smooth muscle activity under hypoxic conditions, mitigating PH severity (Zhou et al. 2022). These findings underscore the complexity of VEGFR-2 regulation across diverse pathologies, highlighting it as a potential therapeutic target not only in cancer but also in autoimmune disorders, cerebrovascular disease, and pulmonary hypertension.

## VEGF and VEGFR targeted therapies

Angiogenesis is a harmonious biological process in which pre-existing blood vessels act as templates to synthesize new blood vessels that are highly regulated by VEGF and its receptors (VEGFRs). These molecular entities play crucial roles in normal physiological processes like embryonic development, wound healing, hematopoiesis, and reproductive health. However, they are also involved in pathological conditions, contributing to the development of cancer, inflammation, and abnormal angiogenesis. They also help cancer by providing a continuous supply of nutrients and oxygen and scavenging waste from the tumor cells to allow their proliferation, survival, and metastasis. Researchers have explored the mechanism of these entities, their associated molecular pathways, and the role of every molecule involved in the activation of these entities. This led to the discovery of different VEGF and VEGFR targeted therapies that prevent the binding of VEGF mitogens to VEGF receptors to impede the angiogenic process, which helped us further understand the pathological process mediated by these molecular entities and has revolutionized the treatment landscape for various diseases. These therapies primarily consist of anti-VEGF inhibitors and multi-tyrosine kinase inhibitors that impede the angiogenesis process by targeting VEGF and VEGFRs, as summarized in Table 1. Whereas in Table 2, we have listed numerous investigational drugs that are being explored in clinical trials targeting VEGFRs. These drugs have promising efficacy and safety profiles than previously discovered/approved inhibitors that can be further used to refine and expand the therapeutic arsenal against angiogenesis. These efforts highlight the importance of further research and development in anti-angiogenic therapy to ameliorate efficacy and patient quality of life and reduce side effects.

**Table 1:** List of Anti-VEGF therapies approved by FDA

Company	Drug name	Generic name	Approved year by FDA	Clinical applications and specific therapies
Novartis	Gleevec	Imatinib	2001	Chronic myeloid leukemia, gastrointestinal stromal tumors (GIST)
Genentech	Avastin	Bevacizumab	2004	Metastatic colorectal cancer, non-small cell lung cancer, glioblastoma, renal cell carcinoma, cervical cancer
Eyetech Pharmaceuticals	Macugen	Pegaptanib	2004	Neovascular (wet) age-related macular degeneration
Bayer	Nexavar	Sorafenib	2005	Renal cell carcinoma, hepatocellular carcinoma
Genentech	Lucentis	Ranibizumab	2006	Neovascular (wet) age-related macular degeneration, diabetic macular edema, retinal vein occlusion
Novartis	Votrient	Pazopanib	2009	Renal cell carcinoma, soft tissue sarcoma
Genzyme Corporation	Caprelsa	Vanditanib	2011	Medullary thyroid cancer
Pfizer	Sutent	Sunitnib	2011	Renal cell carcinoma, gastrointestinal stromal tumors (GIST)
Takeda	Iclusig	Ponatinib	2012	Chronic myeloid leukemia, Philadelphia chromosome-positive acute lymphoblastic leukemia
Pfizer	Inlyta	Axitnib	2012	Renal cell carcinoma
Bayer	Stivarga	Regorafenib	2012	Metastatic colorectal cancer, gastrointestinal stromal tumors (GIST), hepatocellular carcinoma
Sanofi and Regeneron	Zaltrap	Ziv-aflibercept	2012	Metastatic colorectal cancer
Chengdu Kanghong Biotech Company	Lumitin	Conbercept	2013	Neovascular (wet) age-related macular degeneration
Bristol Myers Squibb	Pomalyst	Pomalidomide	2013	Multiple myeloma
Boehringer Ingelheim	Ofev and Vargatef	Nintedanib	2014	Idiopathic pulmonary fibrosis, non-small cell lung cancer
Eisai	Lenvima	Lenvatinib	2015	Thyroid cancer, renal cell carcinoma, hepatocellular carcinoma
CTTQ Pharma	Focus V	Anlotinib	2017	Non-small cell lung cancer, soft tissue sarcoma
Novartis	Beovu	Brolucizumab	2019	Neovascular (wet) age-related macular degeneration
Eli Lilly and Company	Cyramza	Ramucirumab	2020	Gastric cancer, non-small cell lung cancer, hepatocellular carcinoma, colorectal cancer
Exelixis Inc	Cabometyx	Cabozantinib	2021	Renal cell carcinoma, hepatocellular carcinoma, differentiated thyroid cancer
AVEO	Fotivad	Tivozanib	2021	Renal cell carcinoma
Pfizer	Lorbrena	Lorlatinib	2021	Non-small cell lung cancer
Hengrui Pharma	Aitan	Revoceranib	2023	Hepatocellular carcinoma
Takeda	Fruzaqla	Fruquintinib	2023	Colorectal cancer
Genentech	Vabysmo	Faricimab	2023	Neovascular (wet) age-related macular degeneration, diabetic macular edema

The information about these drugs is taken from these review articles (Zhang et al. 2023, Wang et al. 2023)

Those drugs are multi-tyrosine kinase inhibitors including VEGF and VEGFRs

**Table 2:** List of Drug being explored in Clinical Trials and Experimental Studies

Company	Drug name	Clinical trial	Clinical applications and PATIENT groups
Akeso Biopharma	AK109	Phase I (NCT04547205)	Solid tumors
Pfizer	TU-68 (Orantinib)	Last Update 2012	Hepatocellular carcinoma
Janssen	JNJ-81201887	Phase I (NCT05811351)	Advanced or metastatic solid tumors
Bristol Myers Squibb	BMS-794833	Phase I (Data not available)	Advanced solid tumors
Bristol Myers Squibb	BMS-777607	Phase II (NCT00605618)	Advanced or metastatic gastric cancer, gastroesophageal junction, or lower esophageal adenocarcinoma
MethylGene	Glesatinib (MGCD-265)	Phase II (NCT02544633)	Non-small cell lung cancer with MET alterations
Ambit Biosciences	BMS-599626 (AC480)	Phase I (Data not available)	Advanced solid tumors
Pfizer	CP-724714	Phase II (Data not available)	HER2-positive advanced or metastatic breast cancer
Amgen	AMG-458	-	Preclinical studies for various cancers
AstraZeneca	Recentin (Cediranib)	Phase II	Recurrent glioblastoma, ovarian cancer, colorectal cancer
GlaxoSmithKline	Experimental Drug (Foretinib)	Clinical Trial Stopped in 2015	Previously studied in papillary renal cell carcinoma and gastric cancer
-	Experimental Drug (lenvatinib)	Ongoing Phase1/2	Various solid tumors, including thyroid cancer and renal cell
Novartis	Experimental Drug (Dovitinib)	Pending FDA approval	Renal cell carcinoma, multiple myeloma, breast cancer
Bristol-Myers Squibb	Brivanib alaninate	Phase 2 (NCT00633789)	Hepatocellular carcinoma
Clovis Oncology	Lucitanib	Open Label Phase I/IIa (NCT02053636)	Advanced solid tumors with FGFR1/2 amplification
Orion Pharma	ODM-203	Phase I/IIa NCT02264418	Advanced solid tumors

The information about these drugs is taken from these review articles (Liu et al. 2022, Zhang et al. 2023, Wang et al. 2023)

Those drugs are multi-tyrosine kinase inhibitors including VEGF and VEGFRs

## Resistance mechanism of VEGFR-2 targeted therapies

VEGFR-2-targeted drugs prevent the interaction of VEGF mitogens with the essential activation domains of the VEGFR-2 receptor to prevent tumor growth and metastasis. However, some patients experience progressive disease as they do not respond well to the anti-VEGF therapy. This is due to the activity of other pathway proteins involved in proangiogenic VEGF signaling, including platelet-derived growth factor (PlGF), HIF, and other proteins. These pathways and protein factors compensate for inhibited VEGFR-2 and promote resistance to VEGF-targeted therapies. There are different resistance mechanisms pursued by tumor cells to attenuate the effects of VEGFR-2-targeted therapies, such as:

### VEGF-axis dependent alterations

Tumor cells tackle the VEGF-targeted therapies by activating other VEGF signaling pathways to develop resistance against them. These include:

#### Upregulation of alternative angiogenic factors:

Various alternative pathways are deployed by human cells to deal with the disturbance caused by the normal signaling pathways. In the case of tumor cells, they adopt the same strategy to develop therapy resistance. Upon exposure to anti-VEGF drugs, tumor cells increase the expression of other proangiogenic factors such as Fibroblast growth factor 1 (FGF-1) and FGF-2 (Brooks et al. 2012), TGF-β (Comunanza and Bussolino 2017), MMPs (Deryugina and Quigley 2010), angiopoietin-1 (Fagiani and Christofori 2013), Angiopoietin-2, Hepatocyte Growth Factor(HGF), Placental Growth Factor(PIGF) (Machado et al. 2023), ephrin-A1-2 (Zhuang et al. 2010), epidermal growth factor (Derynck et al. 1987) and stromal cell-derived factor 1 (SDF-1) (Batchelor et al. 2007). These factors stimulate the proliferation and migration of endothelial cells and promote vessel maturation and stabilization, along with the recruitment of endothelial cells to facilitate angiogenesis. Moreover, inhibition of VEGF mitogens, especially VEGF-A, is compensated by other VEGF mitogens, such as VEGF-C and VEGF-D, by binding to these receptors and activating them (Alitalo et al. 2005). On the other hand, some tumor fibroblasts increase the production of platelet-derived growth factor-C (PDGF-C) (Crawford et al. 2009). These strategies help the tumor cells sustain tumor growth and angiogenesis through alternative pathways independent of VEGF.

#### Hypoxia-induced VEGF expression

Low oxygen concentrations in the tumor microenvironment modulate the overexpression of VEGF in response to anti-VEGF therapy. This causes the tumor cells to activate HIF1α in tumor cells (Forsythe et al. 1996) along with SDF1 and VEGF (Ebos et al. 2007), which attract endothelial progenitor cells. These cells then release more pro-angiogenic cytokines, thus stimulating more angiogenesis.

Another strategy adopted by the tumor cells is to select particular clones upon anti-VEGF therapy to thrive in antiangiogenic therapy-induced hypoxia via inactivation of p53 and allow them to metastasize (Yu et al. 2002). Fewer proangiogenic factors are required by these clones, along with the help of extracellular matrix degradation agents and alternate pathways to sustain their survival, growth, and proliferation, leading to increased tumor invasiveness and metastasis.

Eguchi et al. (2022) described in their review that the efficacy of VEFGR-2 inhibitors like bevacizumab decreases due to the mutations or overexpression of VEGF receptors. Therefore, tumor cells sustain their growth and metastasis along with angiogenic signaling pathways through these alterations. Other reports indicate that tumors induce mutation within endothelial cells and make them cytogenetically abnormal, which can result in conformational changes in the receptors, making these anti-VEGF drugs incapable of binding (Hida et al. 2004).

### Non-VEGF pathways

#### Epithelial-mesenchymal transition (EMT)

Exposure to VEGF-targeting medication results in tumor hypoxia, causing tumor cells to release HIF1α that stimulates the epithelial cells to acquire mesenchymal characteristics, also known as epithelial-mesenchymal Transition (EMT). This leads to the activation of EMT-associated signaling pathways that stimulate the expression of Twist and Snail, Slug, ZEB1/2, TGF-β, Wnt, and Notch, decreasing the expression of E-cadherin and vimentin, fibronectin, leading to tumor invasiveness and growth along with metastasis capabilities (Montemagno and Pages 2020).

#### Involvement of other signaling pathways and circulating nontumor proangiogenic factors

Besides EMT and its associated pathways, Anti-VEGF induced hypoxia increases the circulation of nontumor proangiogenic factors such as VEGF, G-CSF, SDF-1, osteopontin, and stem cell factor, which then activate systemic compensatory mechanisms by activating PlGF, FGF-B, or basic fibroblast growth factor (bFGF) and hepatocyte growth factor (HGF) to sustain angiogenesis (Abdullah and Perez-Soler 2012).

C-met is an important pathway that is widely studied in anti-angiogenic therapy resistance (Organ and Tsao 2011). Hepatocyte Growth Factor (HGF), stimulated in response to anti-VEGF therapy, binds to c-MET, causing c-MET receptor dimerization (Uchikawa et al. 2021). Activated c-MET receptor then recruits and activates different downstream signaling molecules such as Ras, ERK, AKT, and STAT3, which promote cell proliferation, survival, motility, and angiogenesis along with tumor invasiveness and metastasis. These findings have spurred the development of c-MET inhibitors.

### Tumor microenvironment and involvement of various cells

#### Role of bone marrow-derived cells

Tumor hypoxia upregulates the expression of HIF-1α in the tumor microenvironment, which results in the recruitment and expansion of cancer-associated fibroblasts and myeloid cells into the tumor environment (Shojaei et al. 2007). These bone marrow-derived cells induce an immunosuppressive tumor microenvironment by reducing antitumor response and promoting angiogenesis, tumor growth, EMT transition, and metastasis. Tumor microenvironments recruit local stromal cells such as pericytes (Abramsson et al. 2003) and cancer-associated fibroblasts (Dong et al. 2004) that secrete a variety of cytokines and angiogenic factors that can stabilize blood vessels and promote tumor survival and resistance to VEGF inhibitors.

## Strategies to combat VEGF resistance

Drug resistance in VEGFR-2 targeted therapies can be overcome with innovative strategies that aim for both adaptive changes within tumors and primary resistance mechanisms. Some of these strategies are:

### Combination therapy approaches

This approach involves targeting angiogenic pathways or inhibiting downstream signaling pathways. In multi-targeting of angiogenic pathways, including drug-targeting alternative angiogenic pathways such as FGF, PDGF, or angiopoietin pathways, and combining with VEGFR-2 inhibitors, effectively blocks these compensatory mechanisms (Liu et al. 2023). Such combinations (VEGFR-2 inhibitor with FGF/PDGF inhibitors) (Shin et al. 2012), (VEGF-2 and VEGFR3 dual inhibitors) (Liu et al. 2022), are currently being explored in clinical trials. Additionally, research is being conducted on the use of the ALK5/VEGFR2 dual inhibitor TU2218 (Kim et al. 2024) and Anti-Epcam and Anti-VEGFR2 nanobodies (Karami et al. 2023) as potential therapeutic strategies. Other downstream pathways, including MAPK/ERK and PI3 K/Akt, play a critical part in resistance that can be blocked by combining VEGFR-2 targeted drugs with PI3 K or mTOR inhibitors to improve treatment outcomes (Tsuji-Tamura and Ogawa 2016).

### Targeting hypoxia and hypoxia-induced factors

The central factor in rendering resistance to VEGF-targeted drugs is hypoxia-driven HIF activity. Integrating HIF-1α and HIF-2α inhibitors could reduce hypoxia-mediated compensatory angiogenesis that is being developed and tested with VEGFR-2 inhibitors (Ban et al. 2010). Moreover, another strategy is to enhance oxygenation in the tumor’s hypoxic microenvironment can disrupt hypoxia-induced resistance, providing effectivity to VEGFR-2 inhibitors effective in various tumors.

### Targeting the tumor microenvironment

Immune cells in the tumor microenvironment are implicated in developing drug resistance to VEGFR-2-targeted therapies. Therefore, amalgamating immune checkpoint inhibitors (such as PD-1/PD-L1 blockers) with VEGFR-2 inhibitors prevents tumor-associated macrophages and other pro-angiogenic immune cells from supporting resistance (Shigeta et al. 2020) and has shown promise in clinical trials (Chen et al. 2024b). In the tumor microenvironment, by ablating the activity of Myeloid-derived suppressor cells (MDSCs), Tumor-associated macrophages and other immune cells can disrupt the pro-angiogenic influence (Li et al. 2021). Such combinations have been explored recently by combining VEGFR-2 inhibitors with CSF1R-targeting drugs (Kang et al. 2023).

### Improving drug delivery and pharmacokinetics

Inadequate penetration of VEGFR-2 inhibitors in the tumors is also another reason for drug resistance. Utilizing nanoparticles or liposome encapsulation of VEGFR-2 inhibitors can improve penetration into tumors, improve drug targeting (Zahedipour et al. 2023), and achieve higher local drug concentrations. Another technique is to develop sustained drug release of VEGFR-2 inhibitors to regulate effective drug levels inside the tumor microenvironment (Shen et al. 2022). These strategies ensure the co-delivery of multiple drugs, such as VEGFR-2 inhibitors with HIF or PI3 K inhibitors.

## Conclusion and future perspective

VEGFR-2 is considered a primary key regulator of hematopoiesis, angiogenesis, and vasculogenesis implicated during development. The binding of VEGF mitogens onto VEGFR results in the activation of tyrosine kinase receptors on the surface of endothelial cells by stimulating receptor dimerization, trans-autophosphorylation of the tyrosine kinase, and intracellular signaling cascades. This event allows the survival, migration, permeability, and proliferation of vascular endothelial cells. The interaction of VEGF- VEGFR-2 is vital for the VEGFR-2 dimerization, followed by specific orientation or positioning of VEGFR-2 subunits to allow attraction of other molecular factors, binding, phosphorylation, and subunits stabilization. The autophosphorylation of the tyrosine residues in the cytoplasmic domain of VEGFR-2 results in the activation of intracellular signaling such as PLCγ-PKC-Raf-MAPK, TSAd-Src-PI3 K-AKT, Src-Cdc42-p38-MAPK, and PKC-PLCγ-VAV2-Rac1-PAK signaling pathways. These intracellular pathways mediate angiogenesis by regulating endothelial cell proliferation, survival, motility, and vascular permeability. In numerous malignant tumors, retinopathies, blood vessels, and neurological disorders, angiogenesis plays a vital role in their disease progression. Including VEGFR-2 downstream signaling and phosphorylation of the tyrosine kinase domain of VEGFR-2 directs the process of formation of new blood vessels.

The structural elucidation, activation mechanism, and signal transduction of VEGFR-2 have allowed us to under the pathological angiogenesis implicated in these diseases. Moreover, researchers have tried to design drugs targeting the structural subunits of VEGFR-2 and VEGF mitogens to prevent the formation of pathological angiogenesis, which has been approved by the U.S. Food and Drug Administration, and is being exploited for the treatment of angiogenesis-related diseases, and also, new experimental drugs are underway to be approved. These drugs, designed to target the VEGFR-VEGF axis, have failed to halt angiogenesis in different disease conditions due to the intricate nature of the VEGFR-2 structure, neglected proangiogenic molecular factors, and intracellular signaling pathways that lead to therapy resistance. This is due to the expression of other proangiogenic and nontumor proangiogenic factors along with the involvement of some myeloid cells. Therefore, to overcome this resistance barrier and improve the prognosis and treatment response, more effective and multimodal targeting drugs must be developed imminently or combine the concoction of different chemotherapeutic agents with approved drugs to efficiently prevent the pathological angiogenic process.
